# The quality of qualitative research. In memory of Lillemor Hallberg (1942– 2022)

**DOI:** 10.1080/17482631.2022.2153706

**Published:** 2022-12-16

**Authors:** Karin Dahlberg

**Affiliations:** Founding Editor of International Journal of Qualitative Studies on Health and Well-Being

In the beginning of the new millennium, Lillemor Hallberg invited colleagues to a meeting where she revealed her plans of starting a journal for qualitative studies in the area of health sciences. I thought this was an enchanting idea, and even if no other colleagues finally approved of the plans, Lillemor and I decided to go for it. In 2005, Taylor & Francis decided to back us up and in March 2006 we were able to publish the first volume and number of International Journal of Qualitative Studies on Health and Well-being. With the drop stiring the water on the cover we wanted to illustrate that even if we were small, we wanted to make change.

Lillemor and I had the same ambition to emphasize quality in the work with the journal, which also was a main argument for the publisher to invite us. We were critical to the publications that revealed a very low level of scientific awareness and too many shallow findings that should not have been published at all. To do better than that, we recruited an editorial board and reviewers with a high level of competence from all areas of qualitative research. We later on realized that we needed to attach quite direct suggestions of how to improve the articles, i.e., besides being editors, we served as advisers (Hallberg, 2008). By that time, there was no money involved, and we worked for free with really high spirits.

One reason for the well-working collaboration was that both of us had experiences from different kinds of approaches to qualitative research. The two of us became friends when we met at a phenomenology seminar in the early 90s, and then we met again in a course that included phenomenology, hermeneutics and Grounded theory, as well as other approaches to qualitative research. It turned out that in the long run, Lillemor liked Grounded theory, while I was drawn by the continental philosophy and the methodology of phenomenology and hermeneutics.

Besides her work with the new journal, Lillemor served as full professor in Halmstad, with all those duties that accompany such position. Besides that, Lillemor was also a driving force in public health activities, which resulted in several research projects, courses and books. As if this was not enough, she also shared my interest for philosophy of science. Most often, when we met, we talked about how sad it was (and still is) that almost all Swedish universities do not pay attention to the theoretical foundations of scientific work. We thought that all research in the health care area, not only the qualitative, would be endorsed by a higher awareness of the theoretical groundwork that comes with basic courses in theory/philosophy of science. In particular, we argued it should be mandatory in the PhD education. If such development would be, our work with QHW would also be much easier, we agreed. However, to contribute to such education ourselves, we opened up a section for “philosophical papers” and we were happy to read and share so many great contributions by authors from Scandinavia as well as from our international audience, which steadily grew.

We also wanted to contribute to such awareness by our own research. After due review, we published Lillemor’s input to the progress of Grounded theory in QHW (Hallberg, 2006). There she discusses the development of Grounded Theory, from the initial publications by Glaser and Strauss, and their subsequent controversies. Further, she points to the development by Strauss and Corbin, which seems to be the approach that was closest to her own position, but she also includes the more constructivist perspective by Charmaz. One of the interesting aspects in her analysis is whether Glaser´s respectively Strauss´ approaches related to positivism and she also mentions their explicit but, at the same time, vague connection to phenomenology.

Early in the work with QHW we saw papers, from different approaches, presenting findings composed by a number of disparate categories, and more or less reported subjective opinions. As a consequence, Lillemor (Ibid.) emphasizes the significance of knowing how to employ the core category, that hold together the revealed meanings. We argued, that such focus has implications for the quality of the results, and e.g., the power of generalization.

The core category in Grounded Theory has its equivalent in phenomenology in the idea of a meaning structure with its emphasis on the essential meanings, which became one of my contributions, presented in QHW (Dahlberg, 2006). Other strategies to present a cohesive description of the results of analysis can be a description of a comprehensive understanding, a main interpretation, or some themes of meaning where one theme bridges the others.

Another epistemological argument, which also is included in the mentioned articles, is the call for objectivity in terms of openness. In phenomenology, openness is favoured by the researcher being involved in bracketing or bridling, with the aim to slow down the process of understanding in order not to letting one´s pre-understandings operate unnoticed (Dahlberg, 2006). In Grounded theory, the researcher may be careful not to not begin the subsequent analysis too early, i.e., with data from only one or two interviews or observations. Such strategy runs the risk of closing for further and maybe better interpretations (Hallberg, 2006).

These examples are chosen to convey our way of working with QHW. Both articles from the first volume mirror our ambition to strengthen the quality of qualitative research and how we wanted to participate in discussions on evidence. This mission of ours was grounded in our mutual respect of each other´s different competence and experiences, and not least how well her understanding of Grounded Theory worked together with my understanding of phenomenology.

With Lillemor Hallberg´s passing, that era has come to a definite end. However, the last time we met and spoke about QHW, we agreed that the journal is well taken care of and that we were pleased to see that the quality of qualitative research still matters. The sorrow of her death is mixed with strong gratefulness for the years we worked together with International Journal of Qualitative Studies on Health and Well-being.
